# Prognostic significance of the pretreatment prognostic nutritional index in patients with epithelial ovarian cancer

**DOI:** 10.18632/oncotarget.26914

**Published:** 2019-06-04

**Authors:** Naoko Komura, Seiji Mabuchi, Eriko Yokoi, Kotaro Shimura, Mahiru Kawano, Yuri Matsumoto, Tadashi Kimura

**Affiliations:** ^1^ Departments of Obstetrics and Gynecology, Osaka University Graduate School of Medicine, Suita, Osaka 565-0871, Japan

**Keywords:** ovarian cancer, prognostic nutritional index, clinical stage, survival, prognostic factor

## Abstract

**Objective:**

We retrospectively investigated the prognostic significance of the pretreatment prognostic nutritional index (PNI) in patients with epithelial ovarian cancer (EOC) according to the clinical stage.

**Methods:**

The baseline characteristics and clinical outcomes of 308 EOC patients were collected and retrospectively reviewed. PNI was defined as 10 × serum albumin (g/L) + 0.005 × lymphocyte count (per mm^3^) in the peripheral blood. The cut-off value of PNI was defined by time-dependent receiver operating characteristics (ROC) analysis. Univariate or multivariate analysis was conducted to evaluate the association between pretreatment PNI, progression-free survival (PFS), and disease-specific survival (DSS) according to the clinical stage.

**Results:**

The cut-off value of PNI was defined as 44.7 in early-stage patients and 42.9 in advanced-stage patient by ROC analysis, respectively. Although decreased PNI was not associated with short PFS or DSS in early-stage patients, it was significantly correlated with short PFS (p<0.0001) and DSS (p<0.0001) in advanced-stage patients. In multivariate analysis, decreased PNI was an independent prognostic predictor of recurrence and short survival in advanced-stage patients.

**Conclusion:**

A decreased pretreatment PNI was an independent poor prognostic factor in patients with advanced EOC.

## INTRODUCTION

Epithelial ovarian cancer (EOC) is the 5th leading cause of cancer-related deaths among women and it is estimated that 22,240 new diagnoses and 14,070 deaths will occur in 2018 in the United States [1]. Due to its asymptomatic nature, more than half of patients are diagnosed with advanced-stage diseases. Although most patients respond to the initial treatment of debulking surgery followed by platinum-based chemotherapy, more than 70% develop recurrence within 5 years. Various prognostic factors in EOC patients have been reported: clinical stage, tumor histology, tumor grade, size of residual tumor after initial surgery or platinum resistance [2–4]. However there are limited predictors that can be used to estimate a patient’s survival prior to the initiation of treatment.

The prognostic nutritional index (PNI) was developed in 1984 and originally used for risk assessment of post-surgical complications [5]. PNI has recently attracted attention as an indicator of a poor prognosis in patients with various solid cancers [6–9]. However, to our knowledge, only two studies have investigated the significance of PNI in EOC patients (Table 1) [10, 11]. In 2016, Miao et al. reported that decreased PNI was significantly correlated with shorter PFS (HR, 1.890; 95%CI, 1.396-2.560; p<0.001) and OS (HR, 1.747; 95%CI, 1.293-2.360; p<0.001) in patients with EOC [10]. Although this study included a relatively large number of patients (n=344), the authors did not evaluate the prognostic significance of PNI according to clinical stage [10]. The following year, Zhang et al. showed that decreased PNI was significantly correlated with shorter PFS and OS in advanced-stage EOC patients (Stage III: PFS p<0.001, OS p<0.001, Stage IV: PFS p=0.005, OS p=0.010). In multivariate analysis, they also showed that decreased PNI was an independent predictor of shorter PFS (HR, 1.815; 95%CI, 1.113-2.958; p=0.0017) and OS (HR, 1.699; 95%CI, 1.035-2.789; p=0.0036) only in stage III EOC patients [11]. However, the number of stage I, II and IV EOC patients included in this study was relatively small [11]. Thus, the prognostic significance of PNI in early- or advanced-stage EOC patients remains unclear.

**Table 1:** Summary of studies investigating the significance of PNI in EOC patients

Authors	Year	Number of patients		Cut off value (PNI)	Conclusion	Analyses according to clinical stage	Multivariate analysis
		Early stage	Advanced stage				
Miao et al [10]	2016	168	176	45	Decreased PNI is associated with poor PFS and OS.	No	Yes
Zhang et al [11]	2017	67	170	47.2	Decreased PNI is associated with poor PFS and OS only in stage III OC.	Yes	Yes
Current study	2018	164	144	Early stage 44.7 Advanced stage 42.9	Decreased PNI is associated with poor PFS and DSS only in advanced-stage EOC.	Yes	Yes

Abbreviations: PNI; Prognostic nutritional index, PFS; Progression-free survival, OS; Overall survival, DSS; Disease-specific survival, EOC; Epithelial ovarian cancer.

In the current study, we investigated the prognostic significance of pretreatment PNI in Japanese women with EOC according to the clinical stage.

## RESULTS

### Definition of decreased PNI according to clinical stage

**Figure 1:**
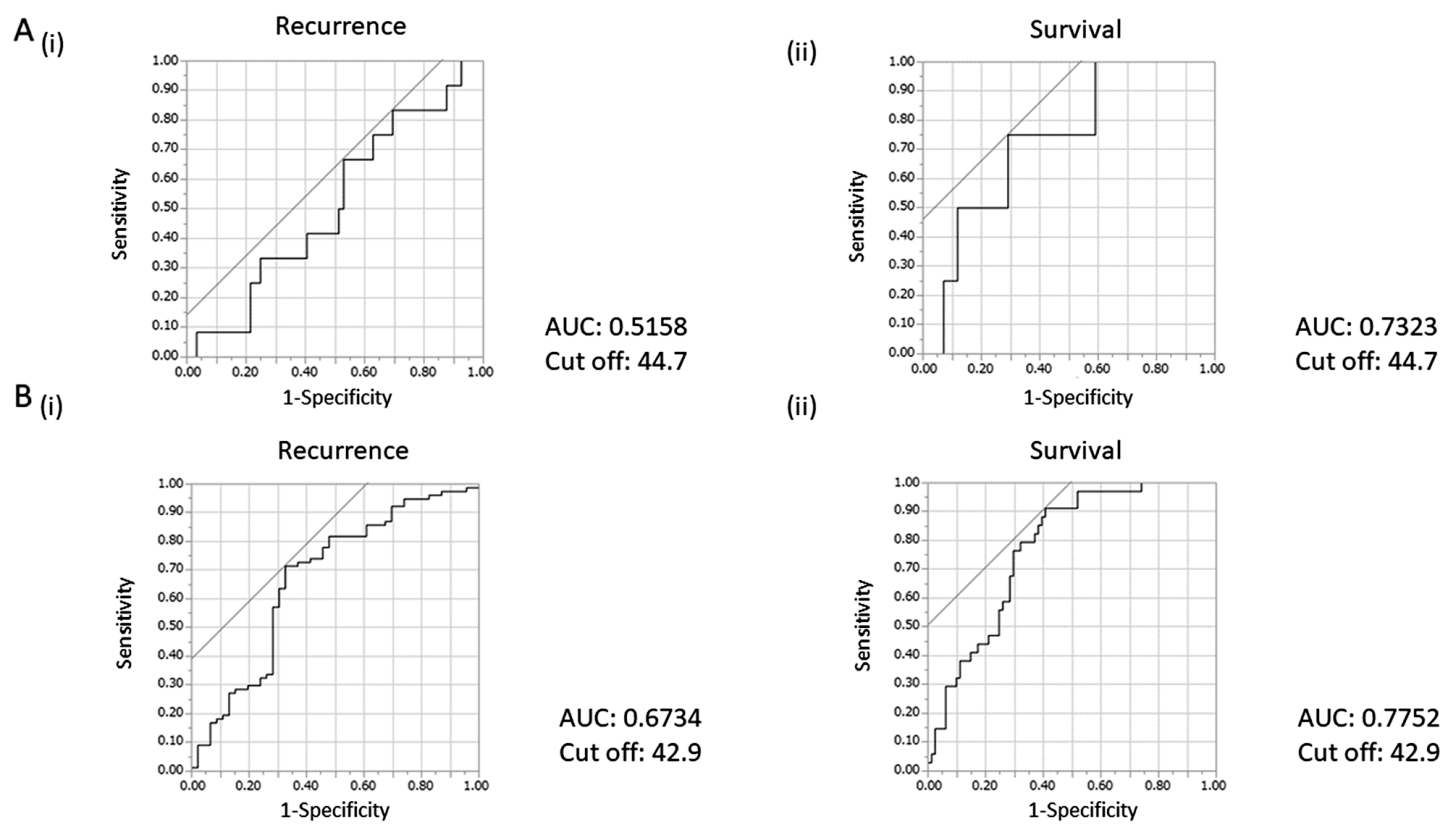
ROC curves for recurrence and survival and cut-off value of prognostic nutritional index (PNI) according to clinical stage **(A)** ROC curves for (i) recurrence and (ii) survival at 2 years for PNI at early stage. **(B)** ROC curves for (i) recurrence and (ii) survival at 2 years for PNI at advanced stage.

ROC curves were described to select the optimal cut-off value for PNI in early- and advanced-stage patients (Figure 1A, 1B). The cut-off values of PNI for recurrence and survival were 44.7 and 42.9 in early- and advanced-stage patients, respectively. The area under the ROC curve (AUC) for predicting recurrence and survival was 0.5158 (95%CI, 0.3546-0.6738) and 0.7323 (95%CI, 0.4505-0.9012), respectively, in early-stage patients, and 0.6734 (95%CI, 0.5627-0.7675) and 0.7752 (95%CI, 0.6790-0.8490), respectively, in advanced-stage patients.

### Prognostic significance of PNI in early EOC patients

**Figure 2:**
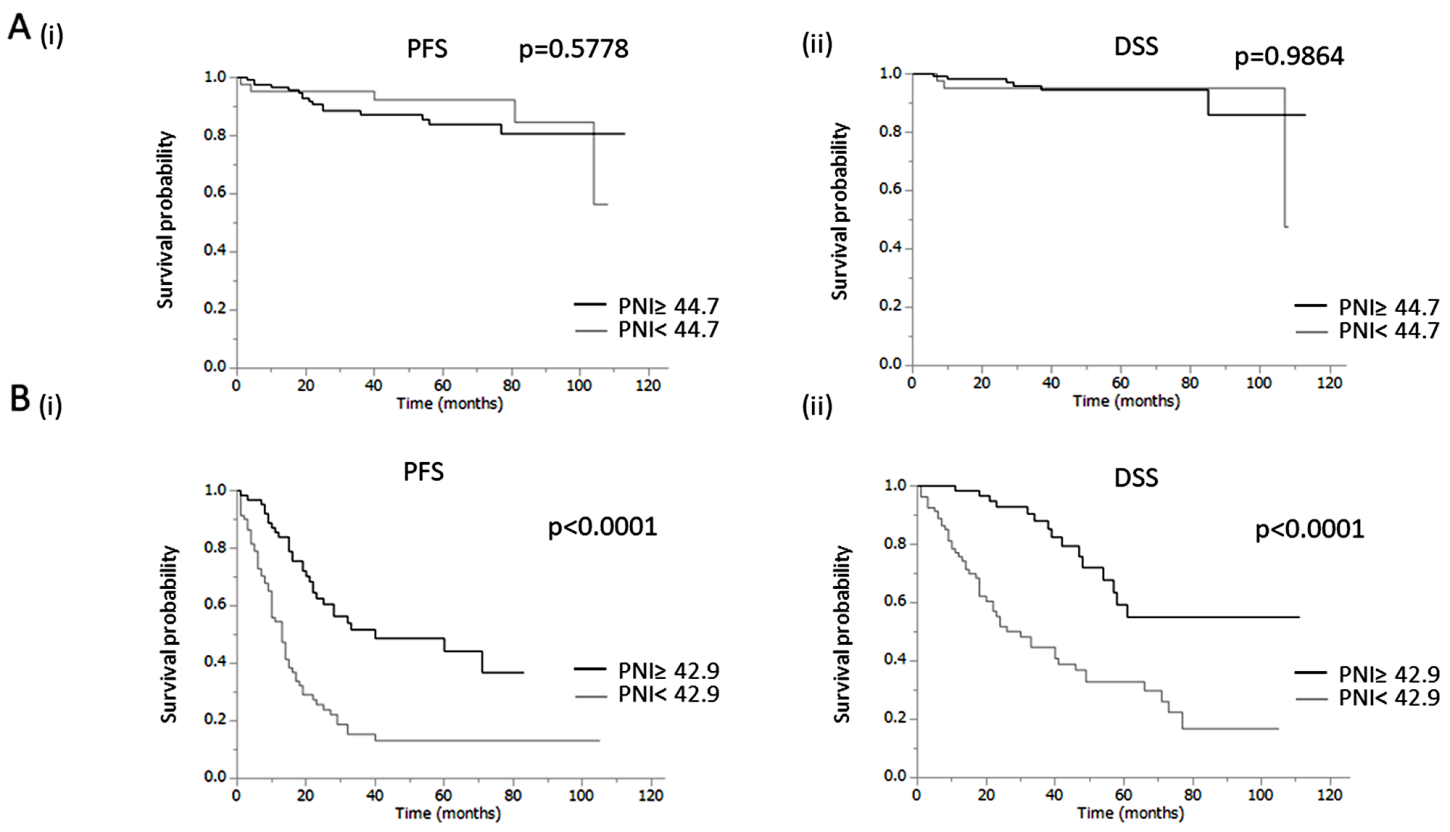
Clinical implications of prognostic nutritional index (PNI) in EOC patients **(A)** Significance of PNI in patients at early stage. (i) Kaplan–Meier estimates of progression-free survival at early stage (PNI: ≥44.7 vs. <44.7). (ii) Kaplan–Meier estimates of disease-specific survival at early stage (PNI: ≥44.7 vs. <44.7). **(B)** Significance of PNI in patients at advanced stage. (i) Kaplan–Meier estimates of progression-free survival at advanced stage (PNI: ≥42.9 vs. <42.9). (ii) Kaplan–Meier estimates of disease-specific survival at advanced stage (PNI: ≥42.9 vs. <42.9).

The characteristics of early-stage EOC patients are shown according to PNI in Table 2. Among the 164 patients with early-stage disease, 44 (26.8%) displayed PNI less than 44.7 at the time of initial diagnosis. Optimal surgery was performed for all early-stage patients. Although there were no differences in age, histology or presence of ascites, decreased PNI was significantly correlated with elevated CA125 (p=0.0159). In the survival analyses, as shown in Figure 2A and Table 3, decreased PNI was not significantly correlated with short PFS (p=0.5778) or DSS (p=0.9864) in early-stage EOC patients. In the multivariate analysis, a decreased PNI was not associated with a shorter PFS (HR, 0.66; 95%CI, 0.21-1.76; p=0.4169) or DSS (HR, 0.77; 95%CI, 0.15-3.04; p=0.7184) in early-stage EOC patients (Supplementary Table 1).

**Table 2:** Clinicopathological characteristics of early-stage patients according to PNI

		All patients (n=164)	PNI< 44.7 (n=44)	PNI≥44.7 (n=120)	
		N	n (%)	n (%)	P-value
Age (years)	<50	64	21 (32.8)	43 (67.2)	0.1665
	≥51	100	23 (23.0)	77 (77.0)	
Histology	Serous	24	8 (33.3)	16 (66.7)	0.0164
	Clear cell	62	22 (35.5)	40 (64.5)	
	Endometrioid	42	10 (23.8)	32 (76.2)	
	Mucinous	24	0	24 (100)	
	Others	12	4 (33.3)	8 (66.7)	
Ascites (ml) ^1^	None	127	30 (23.6)	97 (76.4)	0.2649
	<2000	27	10 (37.0)	17 (63.0)	
	≥2000	2	1 (50.0)	1 (50.0)	
Optimal surgery	Yes	164	44 (26.8)	120 (73.2)	
	No	0	0	0	
CA125 (U/ml)	<500	135	31 (20.0)	104 (80.0)	0.0159
	≥500	29	13 (44.8)	16 (55.2)	

Abbreviations: PNI; Prognostic nutritional index, CA125; carbohydrate antigen 125.

1 The amount of ascites was not indicated for 8 cases.

**Table 3:** Univariate analysis of prognostic factors for progression-free survival and disease-specific survival of early-stage patients based on PNI

		PFS			DSS		
		Univariate analysis			Univariate analysis		
		Hazard ratio	95%CI	P-value	Hazard ratio	95%CI	P-value
Age (years)	<50	1			1		
	≥51	1.40	0.28-1.71	0.4513	1.33	0.37-5.25	0.6626
Histology	Serous	1			1		
	Non serous	1.97	0.64-5.11	0.2159	0.68	0.04-3.73	0.7043
Ascites (ml) ^1^	None	1			1		
	<2000	1.20	0.39-3.12	0.7334	1.35	0.28-5.03	0.6737
	≥2000	NA			NA		
CA125(U/ml)	<500	1			1		
	≥500	3.04	1.20-7.23	0.0202	2.06	0.44-7.40	0.3230
PNI	≥44.7	1			1		
	<44.7	0.72	0.23-1.84	0.5089	0.99	0.21-3.57	0.9847

Abbreviations: CI; confidence interval, CA125; carbohydrate antigen 125, PNI; Prognostic nutritional index, NA; not available.

1 The amount of ascites was not indicated for 8 cases.

### Prognostic significance of PNI in advanced EOC patients

The characteristics of advanced-stage EOC patients are shown according to PNI in Table 4. Among the 144 patients with advanced-stage disease, 81 (56.3%) displayed PNI less than 42.9 at the time of initial diagnosis. Although there were no differences in age or histology, decreased PNI was significantly correlated with large amount of ascites (p<0.0001), a lower optimal surgery rate (p=0.0127) and elevated CA125 (p=0.0004). As shown in Figure 2B, decreased PNI was significantly correlated with a shorter PFS (p<0.0001) and DSS (p<0.0001) in advanced-stage patients. Multivariate analysis showed that in addition to a low optimal surgery rate, decreased PNI was an independent prognostic factor for PFS (Table 5: HR, 2.24; 95%CI, 1.18-4.25; p=0.0139) and DSS in advanced-stage patients (Table 6: HR, 2.85; 95%CI, 1.16-7.11; p=0.0217).

**Table 4:** Clinicopathological characteristics of advanced-stage patients according to PNI

		All patients (n=144)	PNI< 42.9 (n=81)	PNI≥42.9 (n= 63)	
		N	n (%)	n (%)	P-value
Age (years)	<50	37	21(56.8)	16 (43.2)	0.9425
	≥51	107	60 (56.1)	47 (43.9)	
Histology	Serous	92	50 (54.3)	42 (45.7)	0.1349
	Clear cell	16	12 (75.0)	4 (25.0)	
	Endometrioid	11	3 (27.3)	8 (72.7)	
	Mucinous	4	3 (75.0)	1 (15.0)	
	Others	21	13 (61.9)	8 (38.1)	
Ascites (ml) ^1^	None	60	16 (26.7)	44 (73.3)	<0.0001
	<2000	38	27 (71.1)	11 (29.0)	
	≥2000	28	25 (89.3)	3 (10.7)	
Optimal surgery ^2^	Yes	76	33 (43.4)	43 (56.6)	0.0127
	No	55	36 (65.5)	19 (34.5)	
CA125 (U/ml)	<500	63	25 (39.7)	38 (60.3)	0.0004
	≥500	81	56 (69.1)	25 (30.9)	

Abbreviations: PNI; Prognostic nutritional index, CA125; carbohydrate antigen 125.

1 The amount of ascites was not indicated for 18 cases.

2 Surgery was performed in 131 cases.

**Table 5:** Univariate/ Multivariate analysis of prognostic factors for progression-free survival of advanced-stage patients based on PNI

		Univariate analysis			Multivariate analysis		
		Hazard ratio	95%CI	P-value	Hazard ratio	95%CI	P-value
Age (years)	<50	1					
	≥51	1.41	0.87-2.41	0.1667			
Histology	Serous	1					
	Non serous	0.99	0.65-1.55	0.9783			
Ascites (ml) ^1^	None	1			1		
	<2000	1.63	0.97-2.72	0.0648	1.09	0.57-2.07	0.7983
	≥2000	2.08	1.15-3.67	0.0169	1.16	0.52-2.54	0.7177
Optimal surgery ^2^	Yes	1			1		
	No	2.40	1.53-3.76	0.0001	2.02	1.22-3.34	0.0065
CA125(U/ml)	<500	1					
	≥500	1.47	0.97-2.26	0.0677			
PNI	≥42.9	1			1		
	<42.9	2.92	1.89-4.60	<0.0001	2.24	1.18-4.25	0.0139

Abbreviations: CI; confidence interval, CA125; carbohydrate antigen 125, PNI; Prognostic nutritional index.

1 The amount of ascites was not indicated for 18 cases.

2 Surgery was performed in 131 cases.

**Table 6:** Univariate/ Multivariate analysis of prognostic factors for disease-specific survival of advanced-stage patients based on PNI

		Univariate analysis			Multivariate analysis		
		Hazard ratio	95%CI	P-value	Hazard ratio	95%CI	P-value
Age (years)	<50	1					
	≥51	1.46	0.80-2.86	0.2254			
Histology	Serous	1					
	Non serous	0.63	0.38-1.04	0.0705			
Ascites (ml) ^1^	None	1			1		
	<2000	1.42	0.72-2.77	0.3060	0.76	0.30-1.92	0.5613
	≥2000	2.68	1.33-5.33	0.0067	1.69	0.63-4.74	0.2990
Optimal surgery ^2^	Yes	1			1		
	No	2.70	1.56-4.76	0.0004	2.53	1.30-5.03	0.0065
CA125(U/ml)	<500	1					
	≥500	1.27	0.77-2.14	0.3449			
PNI	≥42.9	1			1		
	<42.9	3.74	2.14-6.93	<0.0001	2.85	1.16-7.11	0.0217

Abbreviations: CI; confidence interval, CA125; carbohydrate antigen 125, PNI; Prognostic nutritional index.

1 The amount of ascites was not indicated for 18 cases.

2 Surgery was performed in 131 cases.

## DISCUSSION

In the current study, we showed that PNI was an independent prognostic factor for short PFS and DSS in advanced-stage EOC patients. We also demonstrated that PNI is not a prognostic indicator in early-stage EOC patients.

Including ours, three studies have investigated the significance of PNI in EOC patients. As shown in Table 1, PNI was found to be an independent prognostic indicator in EOC patients in all of these studies. Of note, 2 studies, including ours, that evaluated the prognostic significance of PNI according to clinical stage suggested that PNI is an independent prognostic indicator in advanced-stage EOC patients. In both studies, however, PNI did not provide any prognostic information for early-stage EOC patients [10, 11]. Collectively, these results strongly indicate that PNI can serve as a prognostic indicator only in advanced-stage EOC patients.

PNI is calculated by the serum albumin and absolute lymphocyte counts (ALC). Albumin is the most abundant plasma protein in humans. Low serum albumin reflects the nutritional status of patients or an increased leakage of albumin into the extravascular space, which results in hypoalbuminemia, pleural effusion, or edema. Thus, hypoalbuminemia is more frequently observed in advanced- than in early-stage EOC patients. Hypoalbuminemia may also reflect the inflammatory activity induced by cytokines such as interleukin-6 (IL-6) or tumor necrosis factor [12]. Moreover, it has been reported that inflammation can also decrease ALC. In mice, IL-6 was demonstrated to inhibit lymphopoiesis, leading to the increased production of myeloid cells [13]. In fact, decreased ALC significantly correlates with increased serum levels of IL-6 or IL-2 in patients with soft tissue sarcoma [14]. In the current study, decreased PNI was observed in both early- and advanced-stage EOC patients. We speculate that cytokine production (i.e., IL-6) by EOC cells causes both hypoalbuminemia and decreased ALC, leading to decreased PNI. In the current study, early-stage patients had a significantly higher PNI than advanced-stage patients (average PNI; 46.5 versus 40.3, p<0.0001). Accordingly, decreased PNI was more frequently observed in advanced- compared with early-stage patients (56.3% vs. 26.8%, respectively, p<0.001). A decreased PNI in advanced-stage EOC patients has also been reported in previous studies [11]. This may be explained by the increased production of cytokines from larger advanced tumors and the increased leakage of albumin from the highly-developed tumor vasculatures. Although previous studies employed only one cut-off value for both early- and advanced-stage patients [10, 11], we believe that the optimal cut-off value of PNI should be determined according to clinical stages. As shown in Figure 1 and Table 1, we determined the cut-off values of PNI in early- and advanced-stage patients separately from ROC curves in the current study (44.7 in early-stage and 42.9 in advanced-stage patients). As the optimal PNI cut-off values were employed for both early- and advanced-stage EOC patients, we believe that we were able to draw a definitive conclusion regarding the prognostic significance of PNI in EOC patients.

The reason for the poor prognosis of EOC patients with decreased PNI remains unknown. However, both low ALC [15] and hypoalbuminemia [16] have been reported to be associated with shorter survival in patients with EOC, which frequently expresses IL-6 [17]. As there is a positive correlation between tumor-infiltrating lymphocytes and ALC [15, 18] or the albumin level [19], PNI might influence the survival outcome through an immunological mechanism.

The results of the present study may have important clinical implications. First, pretreatment PNI offers individualized survival estimates (Figure 2). Considering the fact that pretreatment PNI does not provide prognostic information for early-stage patients, PNI should only be evaluated in advanced EOC patients. Second, calculation of pretreatment PNI may enable physicians to offer closer follow-up for advanced patients displaying decreased PNI. Third, as PNI calculation requires only low-cost peripheral blood examinations, PNI can be used universally for survival estimation on managing EOC patients.

The limitations of our study need to be addressed. First, this study was conducted at a single institution. Second, this was a retrospective study. We intend to verify our clinical findings in collaborative multi-institutional studies in a prospective setting. The last limitation is the cut-off values of PNI. In the present study, the cut-off values of PNI for recurrence and survival were defined as 44.7 in early-stage patients and 42.9 in advanced-stage patients, respectively. The cut-off values for PNI in previous studies of EOC ranged between 45 and 47.2 (Table 1), which are slightly higher than our cut-off values. The reason for the difference in cut-off values remains unknown; however, the baseline PNI in cancer patients may differ due to ethnicity. Thus, the threshold for PNI need to be further investigated in future studies.

In conclusion, the decreased PNI at the time of initial diagnosis is a predictor of recurrence and shorter survival in advanced EOC patients. Future prospective investigations are warranted for the clinical applications of PNI in EOC patients.

## MATERIALS AND METHODS

### Patients

Permission to proceed with data acquisition and analysis was obtained from the institutional review board of Osaka University Hospital. A list of patients who were diagnosed with epithelial ovarian cancer and treated at Osaka University Hospital between April 2007 and March 2016 was generated from our institutional registry, and the clinical data were analyzed.

### Treatment and post-treatment follow-up

Patients were clinically staged according to the International Federation of Gynecology and Obstetrics (FIGO) staging criteria. The standard procedures for primary cytoreductive surgery consisted of total abdominal hysterectomy (TAH), bilateral salpingo-oophorectomy (BSO), omentectomy, and pelvic and para-aortic lymphadenectomy sampling. In cases with gross unresectable tumors in the peritoneal cavity, retroperitoneal lymphadenectomy was not performed. Postoperative adjuvant chemotherapy consisted of paclitaxel (175 mg/m^2^), and carboplatin (area under the curve: 5) was initiated within 6 weeks of primary cytoreductive surgery when required. Patients with advanced-stage diseases who received platinum-based neo-adjuvant chemotherapy (NAC) instead of primary cytoreductive surgery were also included in this study. Interval debulking surgery was performed whenever possible. The definition of optimal debulking surgery was ≤1 cm being the largest tumor burden remaining at the completion of cytoreduction. Additional surgical procedures such as partial resection of gastrointestinal tract, liver, diaphragm or urinary tract were performed when it was considered necessary to achieve optimal debulking surgery. After the initial treatment, follow-up examinations were conducted by gynecological oncologists at regular intervals in an outpatient clinic.

### Definition of PNI

During the period between the initial diagnosis and start day of the initial treatment, all patients underwent at least 2 blood tests including complete blood counts. PNI was calculated according to the following formula: 10 × serum albumin (g/L) + 0.005 × lymphocyte count (per mm^3^) in the peripheral blood. The cut-off value of PNI was defined based on the maximum Youden index (i.e., sensitivity+specificity-1) in the time- dependent receiver operating characteristics (ROC) curve for recurrence and survival.

### Evaluation of ascites volume

The amount of ascites before treatment was evaluated by five-point method using computed tomography (CT), as reported previously [20, 21]. Briefly, the thickness of ascites in centimeters was measured in three planes such as the bilateral subphrenic space (A and B), the bilateral paracolic space (C and D) and the pre-bladder space (E). The amount of ascites was calculated by following equation; (A+B+C+D+E) x 200 (ml). Amount of ascites could not be evaluated in 26 cases because pretreatment CT were taken at other hospitals.

### Statistical analysis

Continuous data were compared between the groups using Student’s *t*-test, Wilcoxon rank-sum test, or median test. Frequency counts and proportions were compared between groups using the chi-square test or a two-tailed Fisher’s exact test. We performed univariate analysis by comparing the Kaplan–Meier curves for each subgroup with the log-rank test. Progression-free survival (PFS) was defined as the time from the date of the initial surgical procedure or chemotherapy to the date of the first physical or radiographic evidence of disease progression. Disease-specific survival (DSS) was defined as the time from the date of treatment to the date of cancer-related death. P-values of <0.05 were considered significant. All analyses were performed using JMP® software, version 12.0 (SAS Institute, Cary, NC, USA).

## SUPPLEMENTARY MATERIALS TABLE


