# Supplementary material for: Prognostic significance of the pretreatment prognostic nutritional index in patients with epithelial ovarian cancer
Source: Oncotarget. 2019 Jun 4;10(38):3605–13. doi: 10.18632/oncotarget.26914 (PMC6557203; doi:10.18632/oncotarget.26914)
Supplement: Supplementary file 1 [file oncotarget-10-3605-s001.pdf]

Prognostic significance of the pretreatment prognostic nutritional index in patients with epithelial ovarian cancer

SUPPLEMENTARY MATERIALS

Supplementary Table 1: Multivariate analysis of prognostic factors for progression free survival and disease specific survival of early-stage patients based on PNI

		PFS			DSS		
		Multivariate analysis			Multivariate analysis		
		Hazard ratio	95%CI	P-value	Hazard ratio	95%CI	P-value
Age (years)	<50	1			1		
	≥51	1.41	0.56-3.71	0.4696	1,43	0.39-5.83	0.5878
Histology	Serous	1			1		
	Non serous	1.12	0.30-3.44	0.8495	0.43	0.02-2.78	0.4204
Ascites (ml) [1]	None	1			1		
	<2000	1.02	0.31-2.80	0.9745	1.52	0.31-5.91	0.5773
	≥2000	NA			NA		
CA125(U/ml)	<500	1			1		
	≥500	1.52	0.57-4.84	0.4169	2.67	0.53-10.95	0.2142
PNI	≥44.7	1			1		
	<44.7	0.66	0.21-1.76	0.4169	0.77	0.15-3.04	0.7184

Abbreviations: CI; confidence interval, CA125; carbohydrate antigen 125, PNI; Prognostic nutritional index NA; not available.
[1] The amount of ascites was not indicated for 8 cases.
